# Heme sequestration by hemophilin from *Haemophilus haemolyticus* reduces respiratory tract colonization and infection with non-typeable *Haemophilus influenzae*

**DOI:** 10.1128/msphere.00006-24

**Published:** 2024-02-21

**Authors:** Sam Fulte, Brianna Atto, Arianna McCarty, Kadi J. Horn, Jasmina S. Redzic, Elan Eisenmesser, Michael Yang, Robyn L. Marsh, Stephen Tristram, Sarah E. Clark

**Affiliations:** 1 Department of Otolaryngology, University of Colorado School of Medicine, Aurora, Colorado, USA; 2 School of Health Sciences, University of Tasmania, Launceston, Tasmania, Australia; 3 Department of Biochemistry and Molecular Genetics, University of Colorado School of Medicine, Aurora, USA; 4 Department of Pathology, University of Colorado School of Medicine, Aurora, Colorado, USA; 5 Menzies School of Health Research, Charles Darwin University, Darwin, Northern Territory, Australia; University of Michigan, Ann Arbor, Michigan, United States

**Keywords:** *Haemophilus influenzae*, lung infection, nasopharyngeal colonization, *Haemophilus haemolyticus*, hemophilin, hemophore, nutritional immunity, heme, microbiome

## Abstract

Iron acquisition is a key feature dictating the success of pathogen colonization and infection. Pathogens scavenging iron from the host must contend with other members of the microbiome similarly competing for the limited pool of bioavailable iron, often in the form of heme. In this study, we identify a beneficial role for the heme-binding protein hemophilin (Hpl) produced by the non-pathogenic bacterium *Haemophilus haemolyticus* against its close relative, the opportunistic respiratory tract pathogen non-typeable *Haemophilus influenzae* (NTHi). Using a mouse model, we found that pre-exposure to *H. haemolyticus* significantly reduced NTHi colonization of the upper airway and impaired NTHi infection of the lungs in an Hpl-dependent manner. Further, treatment with recombinant Hpl was sufficient to decrease airway burdens of NTHi without exacerbating lung immunopathology or systemic inflammation. Instead, mucosal production of the neutrophil chemokine CXCL2, lung myeloperoxidase, and serum pro-inflammatory cytokines IL-6 and TNFα were lower in Hpl-treated mice. Mechanistically, *H. haemolyticus* suppressed NTHi growth and adherence to human respiratory tract epithelial cells through the expression of Hpl, and recombinant Hpl could recapitulate these effects. Together, these findings indicate that heme sequestration by non-pathogenic, Hpl-producing *H. haemolyticus* is protective against NTHi colonization and infection.

**IMPORTANCE:**

The microbiome provides a critical layer of protection against infection with bacterial pathogens. This protection is accomplished through a variety of mechanisms, including interference with pathogen growth and adherence to host cells. In terms of immune defense, another way to prevent pathogens from establishing infections is by limiting the availability of nutrients, referred to as nutritional immunity. Restricting pathogen access to iron is a central component of this approach. Here, we uncovered an example where these two strategies intersect to impede infection with the respiratory tract bacterial pathogen *Haemophilus influenzae*. Specifically, we find that a non-pathogenic (commensal) bacterium closely related to *H. influenzae* called *Haemophilus haemolyticus* improves protection against *H. influenzae* by limiting the ability of this pathogen to access iron. These findings suggest that beneficial members of the microbiome improve protection against pathogen infection by effectively contributing to host nutritional immunity.

## INTRODUCTION

Non-typeable *Haemophilus influenzae* (NTHi) is a common colonizer of the nasopharynx, from which migration to other tissues can cause disease (1). Prominent NTHi infections include ear infection (otitis media), lung infection (pneumonia), and exacerbations of chronic pulmonary diseases (e.g., chronic obstructive pulmonary disease, protracted bacterial bronchitis, and bronchiectasis) (2–5). While vaccination against encapsulated *H. influenzae* type b (Hib) has reduced the incidence of Hib invasive disease, NTHi infections remain common, particularly among children and the elderly (6, 7). There are currently no approved vaccines targeting NTHi, and antibiotic resistance is emerging within the species (3, 8). NTHi infections and their associated long-term health complications represent a significant global health burden, highlighting the need for novel therapeutic targets.

For NTHi to cause disease, it must first colonize the host epithelium. Survival in this environment is largely dictated by the ability of NTHi to acquire heme, an essential growth factor for this organism. Heme consists of an iron ion contained within a porphyrin ring. In humans, most of the available heme is either bound to proteins including hemoglobin or within heme-containing enzymes and is, therefore, unavailable to microorganisms (9). Levels of free iron ions are extremely low in the host environment due to their toxicity, resulting in their sequestration by proteins including heme, which binds approximately 70% of the iron in the body (10, 11). Iron sequestration is also an important element of nutritional immunity, contributing to baseline protection against pathogens such as NTHi by limiting access to an essential growth factor. NTHi survives in low iron environments by scavenging heme using several TonB-dependent heme uptake systems and by direct import of iron-containing siderophores (12, 13). Therefore, disruption of NTHi heme acquisition presents a potential strategy for reducing NTHi colonization and infection.

Other bacteria in the respiratory tract microbiome, particularly those in the nasopharynx, compete with NTHi for heme, including the closely related commensal *Haemophilus haemolyticus. H. haemolyticus* colonizes the nasopharynx, from which it can be aspirated into the lungs, but unlike NTHi, it is not associated with lung infections or exacerbations of chronic obstructive pulmonary disease (14–16). Select strains of *H. haemolyticus* produce a unique heme-binding protein, or hemophore, called hemophilin (Hpl). Hpl can bind reversibly to reduced ferrous heme or oxidized ferric heme, facilitating heme uptake (17). In the absence of Hpl, *H. haemolyticus* growth is impaired without an alternative source of heme such as hemoglobin, which can be imported by other mechanisms (17). Importantly, Hpl binds heme in a manner that cannot be utilized by NTHi, thus inhibiting NTHi growth *in vitro* (17–20). In humans, colonization with Hpl-expressing *H. haemolyticus* is associated with lower NTHi burdens in the upper airway and a reduced risk of NTHi acquisition, suggesting a beneficial role for Hpl expression (21). Here, we investigated the hypothesis that *H. haemolyticus* reduces NTHi burdens in the upper and lower airway in an Hpl-dependent manner and determined whether recombinant Hpl was sufficient for protection against NTHi colonization and lung infection. We found that in addition to a protective effect for Hpl-producing *H. haemolyticus*, Hpl exposure alone was sufficient to recapitulate several beneficial effects, including lower NTHi adherence to human respiratory tract epithelial cells and reduced NTHi colonization and lung infection in mice.

## RESULTS

### Exposure to *H. haemolyticus* reduces NTHi colonization and lung infection

**Fig 1:**
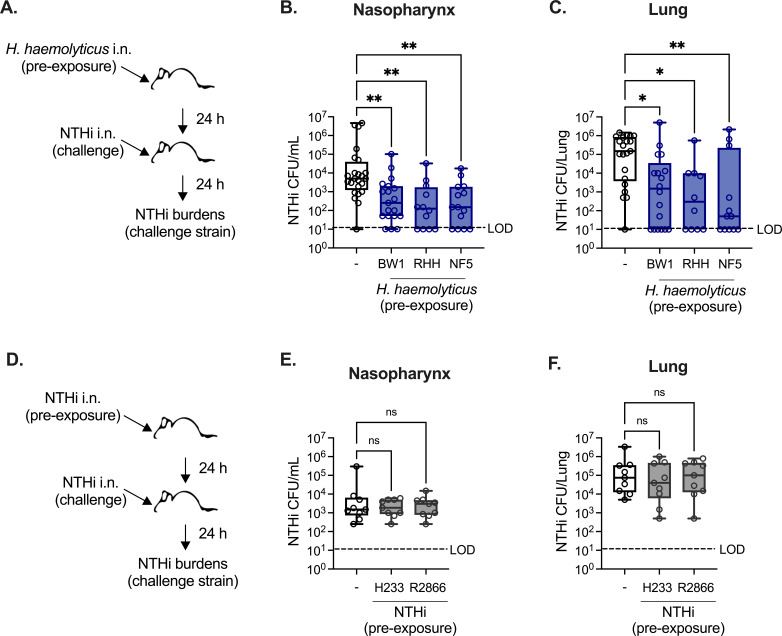
Exposure to *H. haemolyticus*, but not heterotypic NTHi strains, reduces NTHi colonization and infection. (**A**) *H. haemolyticus* pre-exposure and NTHi challenge experimental model. (**B–C**) Burdens of NTHi strain H632 detected in the nasopharyngeal lavage (**B**) and lung tissue (**C**) 24 hours post-infection with 5 × 10^7^–10^8^ CFU/mouse i.n. in wild-type (WT) mice with or without (−) pre-exposure to 10^6^ CFU/mouse i.n. *H. haemolyticus* strains BW1, RHH (RHH122), or NF5 (*n* = 12–24 mice/group). (**D**) Heterotypic NTHi pre-exposure and NTHi secondary challenge experimental model. (**E–F**) Burdens of NTHi strain H632 detected in the nasopharyngeal lavage (**E**) and lung tissue (**F**) in mice with or without pre-exposure to 10^6^ CFU/mouse i.n. NTHi strains H233 or R2866 (*n* = 9 mice/group). Data are pooled from four independent experiments and are displayed as mean ± SEM. ^*^P < 0.05, ^**^P < 0.01, and ^***^P < 0.001, Kruskal–Wallis test with Dunn’s *post hoc* analysis for multiple comparisons. LOD, limit of detection.

To investigate the protective effects of *H. haemolyticus* against NTHi *in vivo*, we utilized a mouse model of NTHi nasopharyngeal colonization and lung infection, which was modified to incorporate intranasal (i.n.) exposure to *H. haemolyticus* prior to NTHi challenge (Fig. 1A). The success of NTHi colonization and lung infection was determined by colony-forming unit (CFU) enumeration in the nasopharyngeal lavage fluid and lung tissue homogenates collected 24 hours post-infection. The use of a streptomycin-resistant NTHi strain (H632) facilitated the selective detection of NTHi. Three different clinical *H. haemolyticus* isolates were evaluated for protective activity, including BW1, RHH122, and NF5, all of which were previously shown to produce the heme-binding protein Hpl (17, 18). Pre-exposure to any of the three *H*. *haemolyticus* strains significantly impaired NTHi colonization of the upper airway, as indicated by reduced nasopharyngeal burdens compared with mice challenged with NTHi alone (Fig. 1B). Similarly, intranasal exposure to each of the *H. haemolyticus* strains BW1, RHH122, or NF5 resulted in significantly lower NTHi burdens in the lungs, compared to mice not exposed to *H. haemolyticus* (Fig. 1C). In some mice, pre-exposure to *H. haemolyticus* resulted in complete clearance of NTHi from the nasopharynx and lungs, as NTHi burdens were below the limit of detection in 15%–33% of mice pre-exposed to *H. haemolyticus*, compared with detectable burdens in almost all mice infected with NTHi alone. Burdens of *H. haemolyticus*, estimated by the subtraction of NTHi growth from total growth on permissive culture plates, were similar between groups (Fig. S1A and B). These data suggest that *H. haemolyticus* reduced the establishment of NTHi colonization and reduced the burden of NTHi in the lungs.

### Heterotypic NTHi infections are not protective against secondary NTHi challenge

To determine whether the reduced NTHi burdens in mice pre-exposed to *H. haemolyticus* were a general effect caused by other non-typeable *Haemophilus* species, we modified our NTHi infection model to incorporate pre-exposure to heterotypic NTHi isolates prior to challenge with NTHi strain H632 (Fig. 1D). Mice were pre-exposed to the NTHi strains H233 or R2866 prior to secondary challenge with NTHi strain H632. Among these NTHi isolates, only H632 is streptomycin-resistant, enabling the selective enumeration of H632 burdens following secondary challenge at 24 hours post-infection. Unlike pre-exposure to *H. haemolyticus*, primary NTHi infection had no impact on secondary challenge with NTHi, as burdens in the nasopharynx and lung tissue were similar regardless of prior NTHi exposure (Fig. 1E through F). Together, these findings indicate that heterotypic protection is not a general feature of *Haemophilus* species and highlight a distinct protective effect for *H. haemolyticus* against NTHi colonization and lung infection.

### Hpl expression facilitates *H. haemolyticus*-mediated protection against NTHi infection

**Fig 2:**
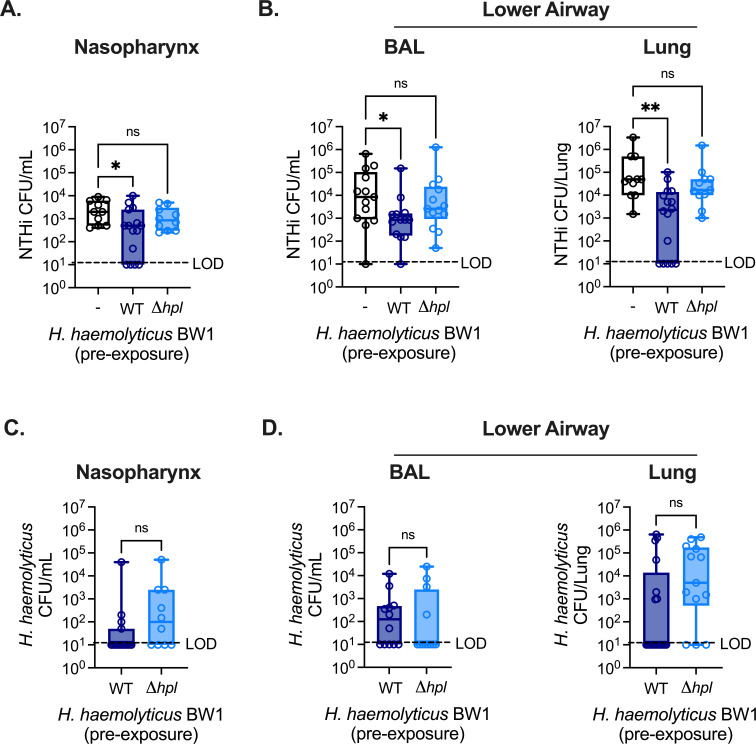
Hpl expression facilitates *H. haemolyticus*-mediated protection against NTHi infection. (**A–B**) Burdens of NTHi strain H632 detected in the nasopharyngeal lavage (**A**) and lower airway BAL and lung tissue (**B**) at 24 hours post-infection with 10^8^ CFU/mouse i.n. in WT mice with or without (−) i.n. pre-exposure to 10^6^ CFU/mouse *H*. *haemolyticus* strain BW1 with intact Hpl expression (WT) or *Δhpl* BW1 (*n* = 10–15 mice/group). (**C–D**) Burdens of *H. haemolyticus* detected in the nasopharyngeal lavage (**C**) and lower airway BAL and lung tissue (**D**) of mice from (A–B). Data are pooled from four independent experiments and are displayed as mean ± SEM. ^*^P < 0.05, ^**^P < 0.01, Kruskal–Wallis test with Dunn’s *post hoc* analysis for multiple comparisons.

*H. haemolyticus* production of the heme-binding protein Hpl is associated with reduced NTHi growth in co-cultures *in vitro* (19), suggesting that Hpl expression may contribute to the protective effect of *H. haemolyticus* against the establishment of NTHi colonization and lung infection *in vivo*. To address this question, we next compared the effect of pre-exposure to the *H. haemolyticus* WT strain BW1 with an Hpl-deficient version of this strain, *Δhpl* BW1. Mice were challenged with NTHi 24 hours post-exposure to *H. haemolyticus* WT BW1 or *Δhpl* BW1, and NTHi burdens were enumerated 24 hours post-infection. In contrast to *H. haemolyticus* WT BW1, which was associated with significantly lower NTHi burdens in the nasopharynx and lungs of pre-exposed mice, the Hpl-deficient BW1 strain had no impact on NTHi colonization or lung infection, suggesting a loss of the protective effect (Fig. 2A and B). As before, several mice pre-exposed to *H. haemolyticus* WT BW1 had NTHi burdens below the limit of detection in the nasopharynx and lung tissue, while this was not the case for any mice pre-exposed to *Δhpl* BW1. In the lower airway, NTHi burdens were significantly reduced in both lung tissue and bronchoalveolar lavage (BAL) fluid of mice pre-exposed to *H. haemolyticus* WT BW1, compared with mice challenged with NTHi alone or Hpl-deficient BW1. Importantly, nasopharynx and lung burdens of WT and *Δhpl* BW1 *H. haemolyticus* were similar between groups, suggesting that enhanced protection against NTHi colonization and infection was not due to increased growth or survival of WT BW1 *in vivo* (Fig. 2C and D). In mice exposed to *H. haemolyticus* WT BW1, but not *Δhpl* BW1, *hpl* expression was detected in the nasopharynx, BAL, and lung tissue (Fig. S2A). These data suggest that Hpl is required for the protective benefit of *H. haemolyticus* exposure in the upper and lower airway against the establishment of NTHi colonization and infection.

### *H. haemolyticus* reduces NTHi growth and adherence to human airway epithelial cells in an Hpl-dependent manner

**Fig 3:**
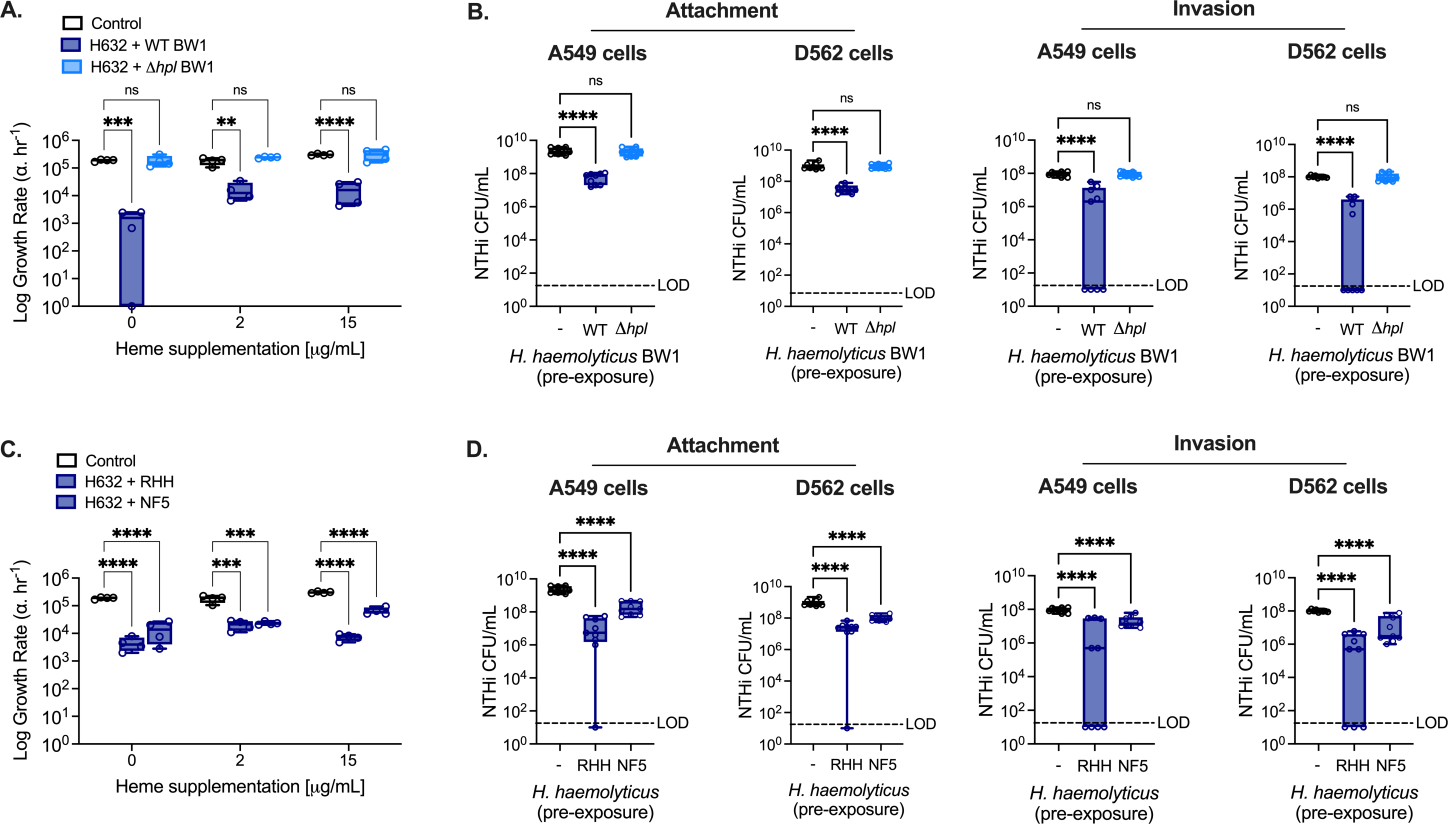
*H. haemolyticus* reduces NTHi growth and adherence to human airway epithelial cells in an Hpl-dependent manner. (**A**) Growth rate of NTHi strain H632 represented as the log change in bacterial density during exponential phase per hour, α·hr^−1^, in cultures co-inoculated with *H. haemolyticus* strain WT BW1 or *Δhpl* BW1. Cultures were performed under minimal heme conditions with or without supplementation as indicated (*n* = 3 independent experiments). (**B**) Attachment and invasion of NTHi strain H632 to human respiratory tract epithelial cell lines, A549 cells and D562 cells, with or without 4-hour pre-exposure to *H. haemolyticus* strain WT BW1 or *Δhpl* BW1 (*n* = 3 independent experiments). (**C**) Growth rate of NTHi strain H632 as in (**A**), in cultures co-inoculated with *H. haemolyticus* strains RHH (RHH122) or NF5 (*n* = 3 independent experiments). (**D**) Attachment and invasion of NTHi strain H632 to A549 cells and D562 cells as in (**B**) with or without 4-hour pre-exposure to *H. haemolyticus* strains RHH (RHH122) or NF5 (*n* = 3 independent experiments). Data are displayed as the mean of two technical replicates per condition for each independent experiment (biological replicate) (**A, C**) or median of three technical replicates per condition for each independent experiment (biological replicate) (**B, D**) ±SEM. ^*^P < 0.05, ^**^P < 0.01, ^***^P < 0.001, and ^****^P < 0.0001, two-way ANOVA with Dunnett’s *post hoc* analysis for multiple comparisons (**A, C**), or Kruskal–Wallis test with Dunn’s *post hoc* analysis for multiple comparisons (**B, D**).

Hpl is secreted by *H. haemolyticus* in broth cultures and following attachment to human airway epithelial cells (19). The direct effect of Hpl production by *H. haemolyticus* on NTHi growth was investigated using the same strain combinations evaluated *in vivo* in a simplified co-culture system *in vitro*. In growth media with minimal heme, co-culture with the Hpl-producing *H. haemolyticus* strain WT BW1 significantly reduced the growth rate of NTHi strain H632, while NTHi growth was unaffected in the presence of the Hpl-deficient strain *Δhpl* BW1 (Fig. 3A). Significantly lower NTHi growth rates were also observed when cultures were supplemented with 15 µg/mL heme, but to a lesser extent than under more heme-limited conditions.

We next compared the effect of Hpl expression on NTHi attachment and invasion of human respiratory tract epithelial cells following exposure to *H. haemolyticus* for 4 hours. These conditions were previously determined to be sufficient for maximal attachment of *H. haemolyticus* to A549 and D562 cells (20). In both A549 and D562 cells, pre-exposure to *H. haemolyticus* WT BW1 significantly reduced attachment of NTHi strain H632, while this effect was absent following pre-exposure to *Δhpl* BW1 (Fig. 3B). NTHi invasion of epithelial cells, measured in separate cultures following gentamicin treatment to kill extracellular bacteria, was also significantly reduced in cultures pre-exposed to WT BW1, but not *Δhpl* BW1. In some cultures, pre-exposure to *H. haemolyticus* WT BW1 resulted in NTHi burdens below the limit of detection, suggesting complete ablation of NTHi invasion.

The impact of *H. haemolyticus* on the growth rate of NTHi strain H632, as well as the attachment and invasion of respiratory tract epithelial cells, was evaluated for other Hpl-expressing *H. haemolyticus* strains, including RHH122 and NF5. As with WT BW1, co-culture with either RHH122 or NF5 Hpl-sufficient *H. haemolyticus* isolates resulted in significantly reduced NTHi growth rates (Fig. 3C). Pre-exposure to RHH122 or NF5 also significantly impaired NTHi attachment and invasion of human respiratory tract epithelial cells (Fig. 3D). For all three *H*. *haemolyticus* strains WT BW1, RHH122, and NF5, *hpl* expression in the respiratory tract epithelial cell cultures was confirmed by qPCR, whereas expression was absent from *Δhpl* BW1 cultures (Fig. S2B). These observations are consistent with a direct role for Hpl expression in *H. haemolyticus*-mediated protection against NTHi infection, as Hpl was required for reduced NTHi growth as well as attachment and invasion of human respiratory tract epithelial cells in the presence of *H. haemolyticus*.

### Recombinant Hpl is sufficient to reduce NTHi burdens *in vivo* and decrease adherence to human respiratory tract epithelial cells

**Fig 4:**
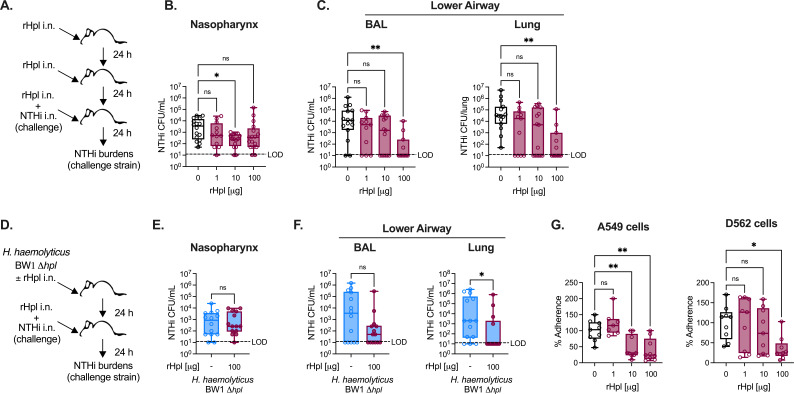
Recombinant Hpl is sufficient to reduce NTHi burdens *in vivo* and decrease adherence to human respiratory tract epithelial cells. (**A**) rHpl exposure and NTHi strain H632 challenge experimental model. Mice were treated with three doses of rHpl at 24-hour intervals prior to NTHi infection. (**B–C**) Burdens of NTHi detected in the nasopharyngeal lavage (**B**) and lower airway BAL and lung tissue (**C**) at 24 hours post-infection with 10^8^ CFU/mouse i.n. with rHpl pre-exposures at the indicated doses (*n* = 11–15 mice/group). (**D**) *H. haemolyticus* strain *Δhpl* BW1 pre-exposure with or without rHpl prior to NTHi strain H632 challenge experimental model. (**E–F**) Burdens of NTHi detected in the nasopharyngeal lavage (**E**) and lower airway BAL and lung tissue (**F**) at 24 hours post-infection with *H. haemolyticus* strain *Δhpl* BW1 and rHpl pre-exposures as indicated (*n* = 14 mice/group). (**G**) Percent adherence of NTHi to human respiratory tract epithelial cell lines, A549 cells and D562 cells, with or without 4-hour pre-exposure to rHpl as indicated (*n* = 3 independent experiments, conducted with three technical replicates per condition). Data are pooled from three to four independent experiments and are displayed as mean ± SEM. ^*^P < 0.05, ^**^P < 0.01, Kruskal–Wallis test with Dunn’s *post hoc* analysis for multiple comparisons (**B–C**), Mann-Whitney *U* test (**E, F**), and one-way ANOVA with Dunnett’s *post hoc* analysis for multiple comparisons (**G**).

To investigate whether Hpl is sufficient for enhanced defense against NTHi colonization and infection, mice were treated with 1, 10, or 100 µg of recombinant Hpl (rHpl) prior to NTHi infection (Fig. 4A). In the nasopharynx, 10 µg of rHpl significantly reduced NTHi burdens, with mean burdens also trending lower in the mice that received 100 µg rHpl (Fig. 4B). In the lower airway, NTHi burdens in both the BAL and lung tissue were significantly reduced in mice exposed to 100 µg of rHpl, and 55% of the mice had no detectable bacteria in the lungs (Fig. 4C). Thirty-six percent to forty percent of mice exposed to other doses of rHpl also had NTHi lung burdens below the limit of detection. Overall, these data indicate that exposure to rHpl is sufficient to improve protection against NTHi colonization and lung infection.

To assess whether the deficit in protection against NTHi for the *Δhpl* BW1 strain of *H. haemolyticus* is due to the lack of *hpl* expression, we performed a rescue experiment with rHpl. Mice were pre-exposed to *Δhpl* BW1 with and without 100 µg rHpl, followed by NTHi challenge (Fig. 4D). At 24 hours post-infection, NTHi burdens were similar in the nasopharynx between groups, suggesting that rHpl was not sufficient to restore protection against NTHi establishment in the upper airway (Fig. 4E). However, NTHi burdens were significantly reduced in the lung tissue of mice that received rHpl in addition to *Δhpl* BW1, with NTHi only detected in 45% of mice dosed with rHpl (Fig. 4F). *H. haemolyticus* burdens were similar between groups, suggesting that elevated protection against NTHi lung infection was not due to *H. haemolyticus* outgrowth in the presence of rHpl (Fig. S2C and D). These data suggest that rHpl is sufficient to restore protection against NTHi lung infection in mice exposed to *Δhpl* BW1, highlighting *hpl* expression as the critical beneficial component of the Hpl-producing strain BW1.

The impact of rHpl on NTHi adherence to human respiratory tract epithelial cells was also examined *in vitro*. In these experiments, the percent adherence of NTHi was measured as a combined metric of bacterial attachment and invasion. In both A549 and D562 cells, the addition of rHpl resulted in a significant reduction in the adherence of NTHi H632 (Fig. 4G). The difference in NTHi cell association following pre-exposure to WT versus Hpl-deficient *H. haemolyticus* observed previously (Fig. 3B and D) suggests that total adherence is affected by the level of NTHi surface attachment. Together, these findings indicate that rHpl is sufficient to reduce NTHi adherence *in vitro*, correlating with impaired lung infection following intranasal Hpl exposure *in vivo*.

### Exposure to rHpl does not elevate NTHi infection-associated immunopathology

**Fig 5:**
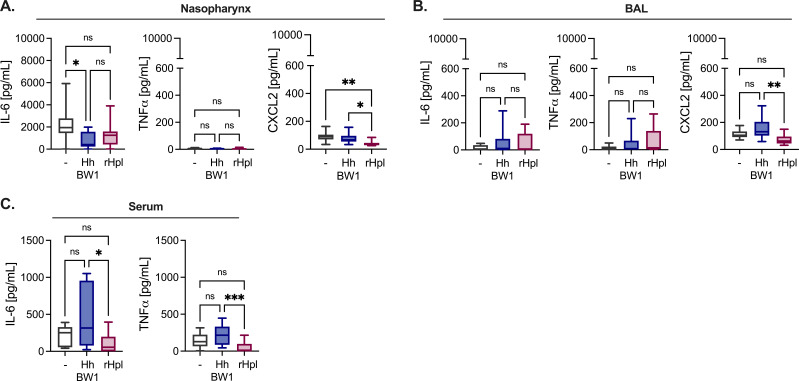
rHpl and *H. haemolyticus* pre-exposures do not increase mucosal or systemic inflammation following NTHi infection. (**A–C**) Cytokines and chemokines detected 24 hours post-infection with NTHi strain H632 10^8^ CFU/mouse i.n. in the nasopharyngeal lavage (**A**), BAL (**B**), and serum (**C**) of mice with or without (−) pre-exposure to *H. haemolyticus* WT BW1 (Hh BW1) or rHpl (*n* = 12 mice/group). Box boundaries indicate the 25th and 75th percentiles, with a horizontal line representing the median and whiskers indicating minimum and maximum values. Data are displayed as the mean ± SEM. ^*^P < 0.05, ^**^P < 0.01, one-way ANOVA with Tukey’s *post hoc* analysis for multiple comparisons.

We next profiled the local and systemic immune response in NTHi-infected mice pre-exposed to either *H. haemolyticus* WT BW1 or rHpl, as both were associated with improved protection against NTHi challenge *in vivo*. A multiplex panel was used to measure cytokine and chemokine concentrations in nasopharyngeal lavage fluid and BAL collected 24 hours post-infection with NTHi. In the nasopharynx, the most abundant analytes were the pro-inflammatory cytokine IL-6 and the chemokines CXCL2, IP10 (CXCL10), and KC (CXCL1) (Fig. 5A; Fig. S3A). Among these, the neutrophil-recruiting chemokine CXCL2 was significantly reduced in mice treated with rHpl, compared with mice infected with NTHi alone or pre-exposed to *H. haemolyticus* (Fig. 5A). Similarly, CXCL2 levels were significantly reduced in the BAL of rHpl-treated mice, compared with mice pre-exposed to *H. haemolyticus* (Fig. 5B). Respiratory tract reductions in CXCL2 were associated with reduced systemic levels of the pro-inflammatory cytokines IL-6 and TNFα, which were significantly lower in the serum of mice treated with rHpl compared with those pre-exposed to *H. haemolyticus* (Fig. 5C). Nasopharyngeal IL-6 was reduced in *H. haemolyticus*-exposed mice compared to untreated mice, though BAL levels of the pro-inflammatory cytokine IFNγ were elevated in this group (Fig. 5A; Fig. S3B). Together, these data suggest that rHpl is associated with a lower pro-inflammatory cytokine response in the serum and reduced mucosal CXCL2. Other analytes including the anti-inflammatory cytokine IL-10 were not significantly altered between groups (Fig. S3A through C).

**Fig 6:**
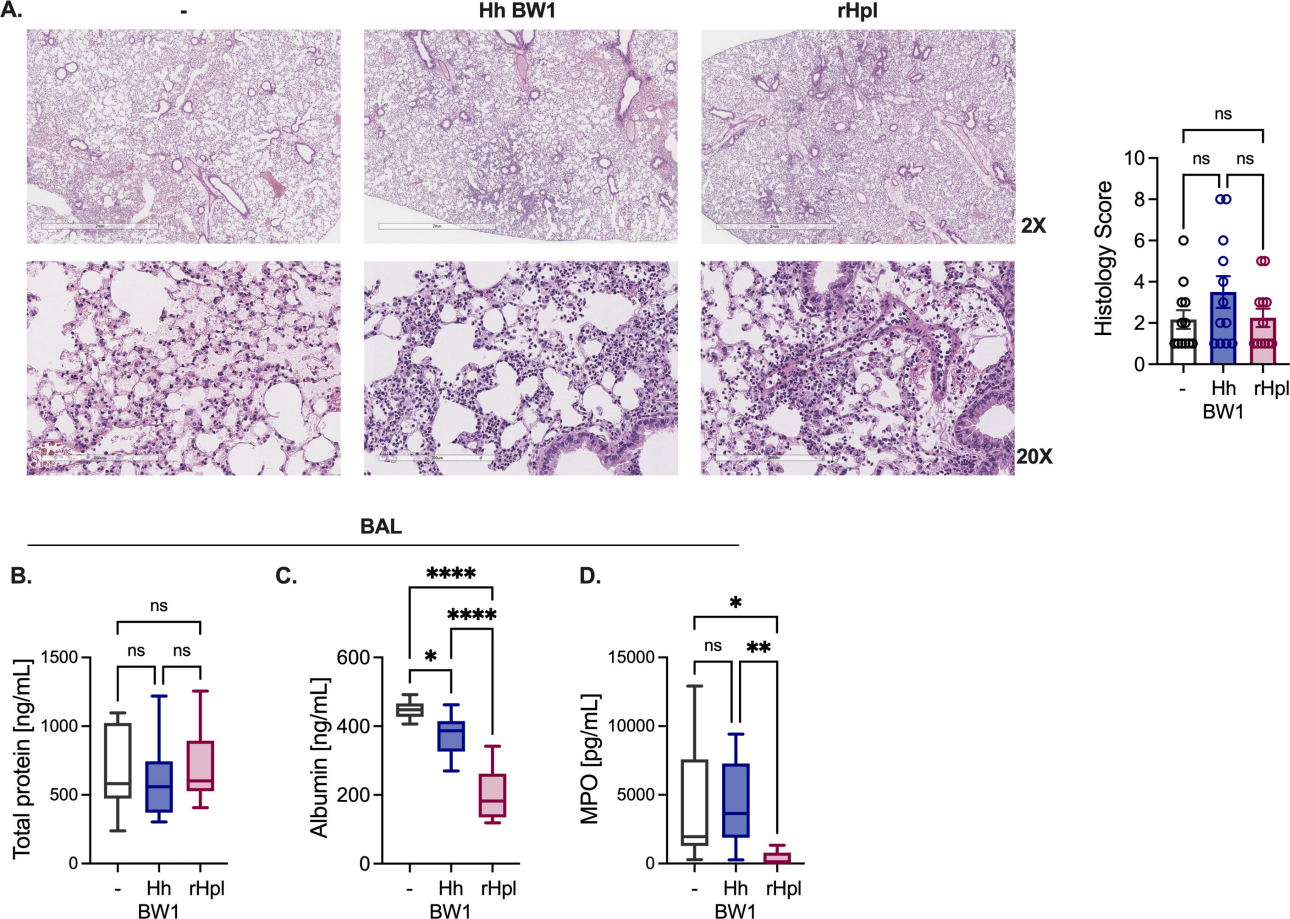
rHpl and *H. haemolyticus* pre-exposures do not exacerbate lung immunopathology following NTHi infection. (**A**) Representative lung tissue sections stained with H&E at 2× and 20× magnification and histopathological scores from lung tissue collected 24 hours post-infection with NTHi strain H632 10^8^ CFU/mouse i.n. with or without (−) pre-exposure to *H. haemolyticus* WT BW1 or rHpl (*n* = 12 mice/group). (**B–D**) Total protein (**B**), albumin (**C**), and MPO (**D**) detected in the BAL of mice from (**A**). Box boundaries indicate the 25th and 75th percentiles, with a horizontal line representing the median and whiskers indicating minimum and maximum values. Data are pooled from three independent experiments and are displayed as the mean ± SEM. ^*^P < 0.05, ^**^P < 0.01, and ^****^P < 0.0001, one-way ANOVA with Tukey’s *post hoc* analysis for multiple comparisons.

Lung immunopathology following NTHi infection was assessed between groups with and without pre-exposure to *H. haemolyticus* BW1 or rHpl using blinded histology scoring of hematoxylin and eosin (H&E) slides prepared from lung tissue (Table S1). Histopathology scores were similar between groups, with the highest scores in NTHi-infected mice pre-exposed to *H. haemolyticus* BW1 (Fig. 6A). Lung epithelial barrier integrity was assessed by measuring total protein and the high molecular weight protein albumin in the BAL. While total BAL protein was unaffected by *H. haemolyticus* or rHpl, the presence of albumin in the BAL was significantly altered (Fig. 6B). BAL albumin was lower in mice pre-exposed to *H. haemolyticus* compared to those infected with NTHi alone, suggesting improved barrier function, and exposure to rHpl was associated with further reduction in albumin leakage (Fig. 6C). In the rHpl-treated group, the level of myeloperoxidase (MPO), a marker of neutrophil activity, was also significantly reduced compared with mice infected with NTHi alone or pre-exposed to *H. haemolyticus* (Fig. 6D). Together, these data indicate that neither *H. haemolyticus* nor rHpl exposures elevated lung immunopathology compared with NTHi infection alone, and instead, some responses including lung MPO and barrier permeability (as measured by albumin levels) were reduced.

## DISCUSSION

The microbiome plays a critical role in defense against infection. However, the lack of species-specific analysis obscures potentially beneficial contributions from commensal bacteria in the same genus as an opportunistic pathogen. The distinction between *H. haemolyticus* and NTHi is particularly difficult, as several phenotypic similarities make conventional microbiology approaches insufficient, necessitating PCR-based differentiation (22). Here, we identify a beneficial role for *H. haemolyticus*, suggesting that microbiome-mediated protection against NTHi may involve non-pathogenic members of the *Haemophilus* genus. Reduced upper airway burdens of NTHi in *H. haemolyticus*-exposed mice suggest that within-genus competition at the primary site of colonization can influence NTHi acquisition. In the lower airway, reduced burdens of NTHi may be affected by *H. haemolyticus* in the lungs, as we observed in the mouse model and has been reported in humans (14–16). However, competition dynamics in the nasopharynx may also influence the amount of NTHi that ultimately reaches the lungs. Of note, in our experiments, NTHi lung burdens were measured downstream of intranasal *H. haemolyticus* or rHpl exposures, as anticipated to occur in humans. Our analysis focused on total differences in NTHi burdens, which was a combined metric including samples with detectable burdens and those where no infection was detected. It is important to consider that successful NTHi infection of cultured cells and mice in the presence of Hpl + *H. haemolyticus* may have involved NTHi adaptation to heme starvation conditions. For example, NTHi under heme-restricted conditions exhibit an extended stationary growth phase with physiological modifications including changes in the biofilm structure as well as metabolic changes associated with intracellular survival and persistence (23, 24). While the impact of such factors on the interactions between NTHi and *H. haemolyticus* remains to be determined, our findings suggest that the expression of the heme-binding protein hemophilin is critical for the protective effect of *H. haemolyticus* against NTHi colonization and lung infection.

Heme uptake by hemophilin appears to be an important heme acquisition strategy for *H. haemolyticus*, whereas heme in this form is unavailable to NTHi (17). We anticipate hemophilin restriction of heme to have a broad impact across NTHi strains, as only a small fraction (<3%) of NTHi genomes encode an Hpl homolog, and it is unclear whether these are functional. *H. haemolyticus* was previously reported to reduce NTHi attachment and invasion of human respiratory tract epithelial cells (25), which we confirm and demonstrate is dependent on Hpl for the Hpl-producing strains included in this study. *In vitro*, recombinant Hpl was sufficient to restrain NTHi adherence to human respiratory tract epithelial cells. *In vivo*, while rHpl had a relatively modest impact on NTHi colonization, a large portion of mice exposed to rHpl failed to establish NTHi infection in the lungs, suggesting that intranasal exposure to recombinant Hpl is sufficient to influence NTHi infection outcomes. These findings highlight heme scavenging by hemophilin as a key point of vulnerability for NTHi, hindering the ability of this pathogen to infect the lower airway.

Iron plays a major role in many microbial processes, including the reduction of oxygen for ATP synthesis, DNA synthesis, and metabolic processes (26). Microbes can acquire iron from the host via receptor-dependent uptake of proteins such as transferrin and heme (27), the production of siderophores (which facilitate iron scavenging), and hemophores (which scavenge heme). Mechanistically, siderophores are small molecule ferric chelators that deliver iron to bacteria in a receptor-dependent manner, meaning that multiple species may import iron-bound siderophores provided they have the appropriate receptor (28). One consequence of this receptor dependence is that siderophore-producing bacteria can induce iron starvation in other bacteria without the matching siderophore receptor. In such cases, iron becomes “locked away” from species lacking the siderophore receptor (29, 30). Bacterial hemophores, which scavenge heme from proteins such as hemoglobin and albumin, are also generally secreted and reenter cells in a receptor-dependent manner (31). In both cases, bacterial uptake of iron or iron-bound heme across the membrane is dependent on the TonB complex (32). Heme uptake is particularly critical for heme auxotrophs such as NTHi, for which it is a growth requirement (11). There are several examples of hemophores made by pathogens, such as the similarly named hemophilin HphA, which shares 39% sequence identity with Hpl and is produced by the opportunistic lung pathogen *Acinetobacter baumannii* (33). Less is understood regarding how commensal bacteria influence the iron acquisition dialog between host and pathogen. Recently, the gut commensal *Bacteroides thetaiotaomicron* was shown to produce a siderophore-binding lipoprotein called XusB, which sequesters the siderophore enterobactin from host capture by lipocalin-2 (34). While enterobactin remains inaccessible to the host when bound to XusB, pathogenic *Salmonella* can recapture the siderophore, taking advantage of *B. thetaiotaomicron* iron sequestration. In contrast, Hpl-bound heme is inaccessible to NTHi (17), resulting in a largely beneficial impact for the host against NTHi colonization.

In addition to “stealing” iron from host proteins, siderophores and hemophores modulate host immune responses (35). For example, the enterobactin upregulates the neutrophil chemokine IL-8 in human respiratory epithelial cells (36) and impedes the bactericidal activity of MPO, which is produced by neutrophils (37, 38). Other siderophores were unable to inhibit MPO activity even at higher concentrations, indicating that this effect is enterobactin-specific (37, 38). In the present study, MPO levels were significantly lower in groups treated with hemophilin; however, it is unclear whether this effect is due to direct interactions or a consequence of reduced NTHi burdens. Regardless, NTHi burdens were reduced, irrespective of MPO activity, in the presence of hemophilin or *H. haemolyticus*, suggesting that this activity was dispensable for Hpl-mediated protection.

Iron limitation is a central host strategy to limit pathogen growth, falling under the umbrella of nutritional immunity. Sequestration of extracellular iron is achieved by iron-binding proteins (including ferritin, transferrin, and lactoferrin) and by capturing iron-containing heme within hemoproteins (39). Iron sequestration is an active process, with immune cells including macrophages involved in iron recycling to limit extracellular iron (40, 41). Additionally, neutrophils responding to active infection produce iron-scavenging proteins including lactoferrin and calprotectin, which also sequesters zinc, manganese, and nickel (42). Neutrophils also produce lipocalin-2, which can bind bacterial siderophores, preventing microbial uptake of iron (43). Pro-inflammatory cytokines released during inflammation also influence iron homeostasis (44). Within the lungs, iron is primarily localized inside the tissue as bound ferritin to prevent oxidative damage (41) with the remaining iron associated with alveolar proteins (45). The total amount of bioavailable iron can be influenced by various factors including air pollution and smoke exposure, with up to a fivefold increase in iron content in the lungs of smokers (46). Iron content in the nasopharynx is thought to be similarly limited (47, 48), though bacterial lysis of host cells can increase heme availability in the form of hemoglobin (48). While not addressed in the current study, there may be additional effects of hemophilin on host immune cell function and by *H. haemolyticus* lysis of epithelial cells, which occurs under some conditions and may influence heme availability for other bacteria (20, 25). The potential indirect effects of Hpl on NTHi attachment to epithelial cells and infection are important areas for future investigation.

Importantly, our findings suggest that *H. haemolyticus*-mediated protection did not exacerbate lung immunopathology. While total protein levels in the BAL were similar, albumin, which is a more specific marker of epithelial damage (49), was significantly reduced, suggesting improved barrier function in *H. haemolyticus* and rHpl-exposed mice. A similar study examined the impact of the murine commensal *Muribacter muris*, which like *Haemophilus* is in the *Pasteurellaceae* family, on NTHi challenge in BALB/c mice co-infected with influenza A virus (50). In this more severe infection model, *M. muris* pre-exposure was associated with reduced NTHi colonization and invasion of the middle ear as well as lower weight loss and clinical scores. *M. muris*-mediated protection correlated with lower inflammation, including reduced IL-6, consistent with our observations with *H. haemolyticus*, as well as lower levels of the chemokine KC (CXCL1) and cytokine IL-1β (50). The mechanism for *M. muris*-mediated protection against NTHi is unclear, though it is unlikely to involve Hpl, as no Hpl homologs have been identified among the small number of published *M. muris* genomes. Regardless, the *M. muris* findings suggest alternative mechanisms for microbiome-mediated protection against NTHi, which could synergize with the beneficial impact of Hpl production by *H. haemolyticus*.

There are several limitations to this study. We did not undertake the full genetic complementation of the *hpl* mutant to confirm the importance of this gene and instead used rHpl reconstitution to demonstrate the recovery of Hpl-mediated protection. Infection experiments were restricted to early time points following NTHi challenge, and it is unclear how *H. haemolyticu*s or hemophilin impacts NTHi colonization and infection dynamics over longer time periods. Further, the therapeutic efficacy of hemophilin will depend on several factors including host toxicity, protein stability, and pharmacokinetics. NTHi infections are also frequently associated with viral co-infection, particularly in the context of otitis media, and it is unclear whether the synergistic effects of viral infection override hemophilin-mediated protection. Finally, while we show a significant impact for hemophilin expression on NTHi adherence to human respiratory tract epithelial cells, additional studies will be necessary to confirm the translational relevance of these findings.

Therapeutic applications of this study beyond treatment with purified hemophilin would include strategies to enhance *H. haemolyticus* colonization as a respiratory tract-directed probiotic approach. Analogous within-genus probiotic applications focus on the use of bacteriocin-producing commensals such as *Streptococcus* strains to treat *Streptococcus pyogenes* (51) and *Staphylococcus lugdunensis*, which reduces nasal carriage of *Staphylococcus aureus* (52). Strategies to enhance colonization of Hpl-producing *H. haemolyticus* strains, particularly for those most vulnerable to NTHi infection, may prove to have a beneficial effect against NTHi-associated disease. However, understanding the implications of Hpl heme sequestration on other pathogenic and non-pathogenic members of the respiratory tract microbiome will be important for the development of such approaches.

## MATERIALS AND METHODS

### Animals

WT (C57BL/6J) mice were purchased from The Jackson Laboratory, stock #000664. Male and female mice aged 7–12 weeks were used for these studies. Animals were maintained by the University of Colorado Office of Laboratory Animal Resources.

### Bacteria

Hpl-producing *H. haemolyticus* respiratory tract isolates RHH122, BW1, and NF5 were used in this study. While NF5 is the lowest Hpl producer among these, *hpl* expression in this strain was still elevated by several logs above that in Δ*hpl* BW1 (Fig. S2B). Δ*hpl* BW1 was created by insertion–deletion replacement of the endogenous *hpl* sequence with a kanamycin cassette, as previously described (17). NTHi strain H632 and clinical isolates H233 and R2866 were a kind gift from Dr. Jeffrey N. Weiser, New York University. NTHi strain H632 is a streptomycin-resistant derivative of the clinical isolate SR332. *H. haemolyticus* and NTHi strains were grown overnight from frozen glycerol stocks on chocolate agar plates (ThermoFisher Scientific) at 37°C with 5% CO_2_. Bacteria were passaged from plates into supplemented brain heart infusion broth (sBHI) containing 2% Fildes Enrichment (ThermoFisher Scientific) and 2 µg/mL β-nicotinamide adenine dinucleotide (NAD, Sigma) and grown for 18 hours at 37°C with shaking at 200 rpm. Plates and broth were supplemented with streptomycin 50 µg/mL (Sigma) for NTHi strain H632.

### NTHi growth rate determination

For co-culture experiments, NTHi and *H. haemolyticus* strains were grown in heme-limited broth to deplete bacterial heme stores without affecting viability. Tryptone soy broth (TSB; Oxoid Ltd.) supplemented with 2% (vol/vol) Vitox (Oxoid Ltd.) and 7.5 µg/mL NAD (sTSB), with 2 µg/mL porcine hematin (ferriprotoporphyrin IX hydroxide, Sigma), was inoculated with strains to an ~0.1 optical density (OD) OD_600_ and incubated for 12 hours at 37°C with shaking (200 rpm). Cultures from 12 hours of growth under these heme-limited conditions were centrifuged (4,000 × *g*, 5 min) and resuspended in pre-warmed supplemented TSB (sTSB) to 1.0 OD_600_ immediately prior to co-culture. NTHi growth rates were determined by the simultaneous addition of heme-limited NTHi (10^8^ CFU) and *H. haemolyticus* (10^7^ CFU) to 5 mL sTSB containing porcine hematin as indicated (0, 2, or 15 µg/mL heme). At timed intervals (0, 4, 6, 8, and 12 hours), 50-µL aliquots were removed for quantitative polymerase chain reaction (qPCR) quantification of bacterial densities; at 8 hours, a 250-µL aliquot was removed and added to 500 µL of RNAprotect (Qiagen) to stabilize RNA for *hpl* expression analysis. NTHi genomic DNA (gDNA) was prepared using thermal extraction, and qPCR for *SiaT* was used for quantification of genome equivalents (bacterial density) against a reference standard, as previously described (19). NTHi growth rates were calculated as the log change in genome equivalents during the exponential phase of growth (16).

### Preparation of recombinant Hpl

rHpl was expressed from the pET28 plasmid previously described (17) encoding a 6xHis-tagged protein. A typical expression comprised 4 L of Luria broth (Difco) at 37°C with kanamycin selection and induced at 0.6 OD_600_ with isopropyl b-D-1-thiogalactopyranoside (Life Technologies) for 3–4 hours. Soluble protein was lysed via sonication and applied to a 20-mL column of Ni-affinity resin (Sigma) in Ni-A buffer (50 mM l Na_2_HPO_4_, pH 7, 500 mM NaCl, 10 mM imidazole) and eluted with Ni-B buffer (50 mM l Na_2_HPO_4_, pH 7, 500 mM NaCl, 400 mM imidazole). Elutions were concentrated using a 10-kDa molecular weight cut off (MWCO) filter (ThermoFisher Scientific) to ~3 mL for further size exclusion chromatography separation using a Superdex-75 column (Cytiva, 120 mL total bed volume) in final buffer (50 mM HEPES, pH 7, 150 mM NaCl). rHpl was quantified by ultraviolet-visible spectrophotometry at 280 nm with extinction coefficient calculation (53). Fractions comprising rHpl were concentrated and stored at −80°C until further use.

### Mouse infection and treatments

Overnight bacterial broth cultures were centrifuged (≥20,000 × *g*) for 10 min to pellet the cells prior to resuspension in phosphate-buffered saline (PBS) at the desired inoculum. The inoculum dose was confirmed by serial dilution for quantification of CFUs. Mice were inoculated intranasally with 25–50 µL of *H. haemolyticus* (10^6^ CFUs total) or rHpl 24 hours prior to NTHi infection under 4% inhaled isoflurane anesthesia. For rHpl, two to three treatments with 1–100 µg per treatment (as indicated) occurred at 24-hour intervals, with the final dose given at the time of NTHi infection. Samples including serum, nasopharyngeal lavage, bronchoalveolar lavage, and lung tissue were collected 24 hours post-NTHi infection. For nasopharyngeal lavages, a tracheal incision was used to flush the nasopharynx with PBS, which was collected through the nares. BAL fluid was obtained by instilling 1-mL PBS into the trachea to flush lower airway bronchioles, after which fluid was retracted for collection. Lungs were removed and homogenized using a Bullet Blender Tissue Homogenizer (Stellar Scientific, Baltimore, MD) in 500-µL PBS. CFUs were calculated following serial dilutions and growth on sBHI or chocolate agar plates supplemented with streptomycin to select for NTHi strain H632. Plates were incubated for 18 hours at 37°C with 5% CO_2_. *H. haemolyticus* CFUs were determined by subtracting CFU counts on streptomycin plates from CFU counts on antibiotic-free plates.

### Cytokine and chemokine analysis

BAL and NL cytokines and chemokines, with the exception of CXCL-2 and MPO, were measured using a LEGENDplex flow cytometry panel (BioLegend), with data analyzed using the LEGENDplex Data Analysis Software Suite (BioLegend). Analytes were detected on an LSR Fortessa X-20 in the University of Colorado Flow Cytometry Shared Resource core (RRID: SCR_022035). BAL CXCL-2 and MPO and serum cytokines were measured by enzyme-linked immunosorbent assay (ELISA) (R&D Systems) with analytes detected on a Synergy HT Microplate Reader (BioTek).

### Human epithelial cell assays

A549 (ATCC #CCL-185) human lung carcinoma epithelial cells and Detroit 562 (D562) human pharyngeal carcinoma epithelial cells (ATCC #CCL-138) were maintained in Ham’s F-12 (Kaighn’s) medium (ThermoFisher Scientific) supplemented with 10% fetal bovine serum (CPS Serum) and 10,000 U/mL penicillin–streptomycin (ThermoFisher Scientific). At confluence, cells were washed with PBS, trypsinized for 5 min at 37°C with 5% CO_2_, washed, and resuspended in F-12 + 10% FBS. Live cells were counted using a hemocytometer after trypan blue staining and seeded into 24-well plates at 10^5^ cells/well and incubated for 48 hours until confluency prior to bacterial challenge. Immediately before bacterial challenge, cells were washed with PBS to remove any non-adherent cells, and media were replaced with fresh F-12 + 10% FBS. For *H. haemolyticus* pre-exposures, cells were treated with bacteria for 4 hours prior to NTHi challenge. Cells were then treated with NTHi in triplicate at the indicated MOI with or without rHpl as indicated, centrifuged for 3 min at 215 × *g*, and then incubated for 4 hours at 37°C with 5% CO_2_. To recover adherent NTHi, cells were washed three times to remove non-adherent bacteria, trypsinized for 15 min at 37°C with 5% CO_2_, and lysed in water. To recover intracellular NTHi (invasion), separate cultures were treated with gentamicin (200 µg/mL, Sigma) for 1 hour following NTHi infection to kill extracellular bacteria prior to trypsinization and lysis. Cell lysates were serially diluted, plated on sBHI with streptomycin, and incubated for 18 hours at 37°C with 5% CO_2_. Total culture CFUs are shown for NTHi attachment. Percent adherence was calculated by dividing total CFUs by CFUs from NTHi incubated in media without cells for the length of the experiment, with values normalized relative to the untreated control group.

### Quantification of *hpl* expression

*H. haemolyticus* expression of *hpl* was quantified by qPCR as previously described (19). Cell lysates following NTHi infection were used to quantify *hpl* mRNA in epithelial cell cultures pre-treated with *H. haemolyticus*. For *in vivo hpl* expression, mice were exposed to either *H. haemolyticus* strain WT BW1 or *Δhpl* BW1 for 6 hours. Samples were prepared from nasopharyngeal lavage fluid, BAL, and lung tissue (homogenized as above) following enrichment for bacteria using a two-step centrifugation process. The first centrifugation step removed host cells (5 min, 500 × *g*) followed by centrifugation for 10 min at ≥20,000 × *g* to pellet bacteria. Briefly, samples were stabilized in RNAprotect Bacteria Reagent (Qiagen) prior to lysate preparation by resuspension of pellets in 100 µL TE buffer (30 mM Tris-Cl, 1 mM EDTA, pH 8.0) with 15 mg/mL lysozyme and 20 µL proteinase K. Following 1-hour lysis at room temperature with shaking at 1,000 rpm, mRNA was extracted using the RNeasy Plus Mini Kit (Qiagen), and cDNA was prepared with the iScript cDNA Synthesis Kit (Bio-Rad). A validated triplex PCR containing the primers and probes for *hpl* detection using Hpl forward (5′-TATTCCTAATGATCCCGCT), Hpl reverse (5′-TCTTTTTTCGCTACCCCT), and an Hpl LNA probe (/5Cy5/AT+CCATTTA+TCGG+CACGTTCT/3IAbRQSp/), with *hpl* expression determined relative to *hypD* as a reference gene. PCR reactions were performed on a CFX96 Touch real-time PCR system (Bio-Rad) with the following steps: 95°C for 3 min (1×), 95°C for 15 sec, and 62°C for 1 min (40×). The expression of *hpl* was calculated relative to that detected in cell cultures or animals exposed to *Δhpl* BW1 *H. haemolyticus*.

### Lung pathology

For histology, lungs were collected in 70% ethanol prior to slide preparation by paraffin embedding and tissue slicing followed by H&E staining by the University of Colorado Research Histology Shared Resource core (RRID: SCR_021994). High-resolution slide images collected using Aperio digital pathology slide scanning were analyzed using ImageScope (Leica Biosystems). Blinded analysis was used to assign histology scores (Table S1). Total BAL protein was measured using a Pierce BCA protein assay kit (ThermoFisher Scientific), and BAL albumin was measured by ELISA (Eagle Biosciences Inc, Fisher Scientific). Analytes were detected on a Synergy HT Microplate Reader (BioTek).

### Statistical analysis

GraphPad (Prism, version 10) was used to conduct statistical analyses. Two-tailed Student’s *t*-tests or one-way ANOVA for multiple comparisons were used for data with normal distributions (Shapiro–Wilk test), and two-tailed Mann–Whitney *U*-tests or Kruskal–Wallis tests for multiple comparisons were used for data with non-Gaussian distributions. *P*-values of <0.05 were considered significant.

### Study approval

All animal studies were approved by the Animal Care and Use Committee of the University of Colorado School of Medicine (protocol #927). The use of biohazardous materials was approved by the Institutional Biosafety Committee (protocol #1418).

## Data Availability

All data supporting the findings of this study are available within the paper and its supplementary files.

## SUPPLEMENTAL MATERIAL

Supplemental Information msphere.00006-24-s0001.pdf Supplemental figures and table.

ASM does not own the copyrights to Supplemental Material that may be linked to, or accessed through, an article. The authors have granted ASM a non-exclusive, world-wide license to publish the Supplemental Material files. Please contact the corresponding author directly for reuse.
