# Supplementary material for: Heme sequestration by hemophilin from Haemophilus haemolyticus reduces respiratory tract colonization and infection with non-typeable Haemophilus influenzae
Source: mSphere. 2024 Feb 21;9(3):e00006-24. doi: 10.1128/msphere.00006-24 (PMC10964412; doi:10.1128/msphere.00006-24)
Supplement: Supplemental Information — Supplemental figures and table. [file msphere.00006-24-s0001.pdf]

SUPPORTING INFORMATION

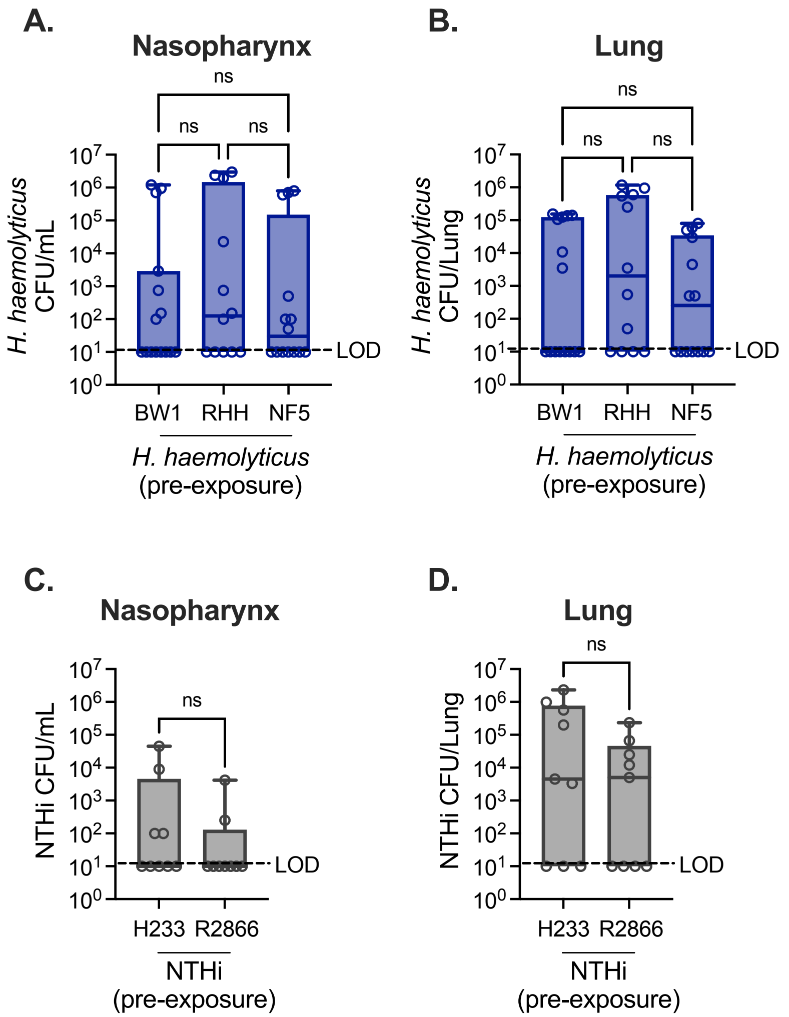

Supplemental Figure 1. Burdens of *H. haemolyticus* and heterotypic NTHi strains in mice challenged with NTHi. A-B Burdens of *H. haemolyticus* strains BW1, RHH122, or NF5 detected in the nasopharyngeal lavage (A) and lung tissue (B) 24 h post-infection with NTHi strain H632 $5x10^7$ - 10^8 CFU/mouse intranasally (i.n.) in WT mice pre-exposed to 10^6 CFU/mouse i.n. *H. haemolyticus* (*n* = 10-24 mice/group). **C-D** Burdens of heterotypic NTHi strains H233 and R2866 detected in the nasopharynx (C) and lung tissue (D) 24 h post-infection with NTHi strain H632 (*n* = 10-15 mice/group). Data are

pooled from 4 independent experiments and are displayed as mean ± SEM. LOD = limit of detection.

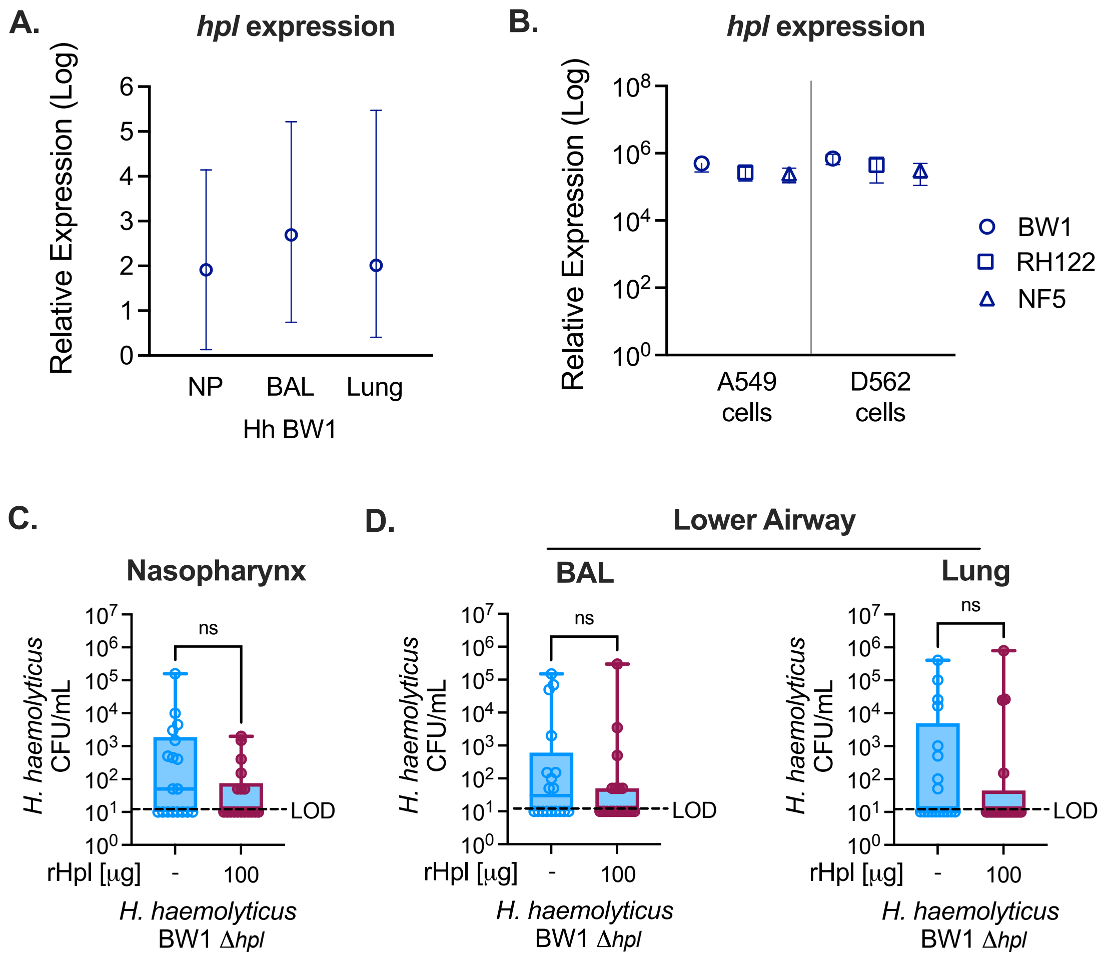

Supplemental Figure 2. *Hpl* expression and *H. haemolyticus* burdens following rHpl treatment in vivo. **A** *H. haemolyticus* expression of *hpl* from the tissues of mice exposed to *H. haemolyticus* strain BW1, relative to expression detected in tissues of mice exposed to *H. haemolyticus* strain *Δhpl* BW1 (*n* = 7-10 mice/group). **B** *H. haemolyticus* expression of *hpl* in respiratory tract epithelial cultures, relative to expression detected in *H. haemolyticus* strain *Δhpl* BW1 (*n* = three biological

replicates). Data are pooled from duplicate mRNA extractions and displayed as mean ± SEM. **C-D** Burdens of *H. haemolyticus* detected in the nasopharyngeal lavage (C) and lower airway BAL and lung tissue (D) at 24 h post-infection with NTHi strain H632 with *H. haemolyticus* strain *Δhpl* BW1 and rHpl pre-exposures as indicated (*n* = 14 mice/group). Data are pooled from 3 independent experiments and are displayed as mean ± SEM. LOD = limit of detection.

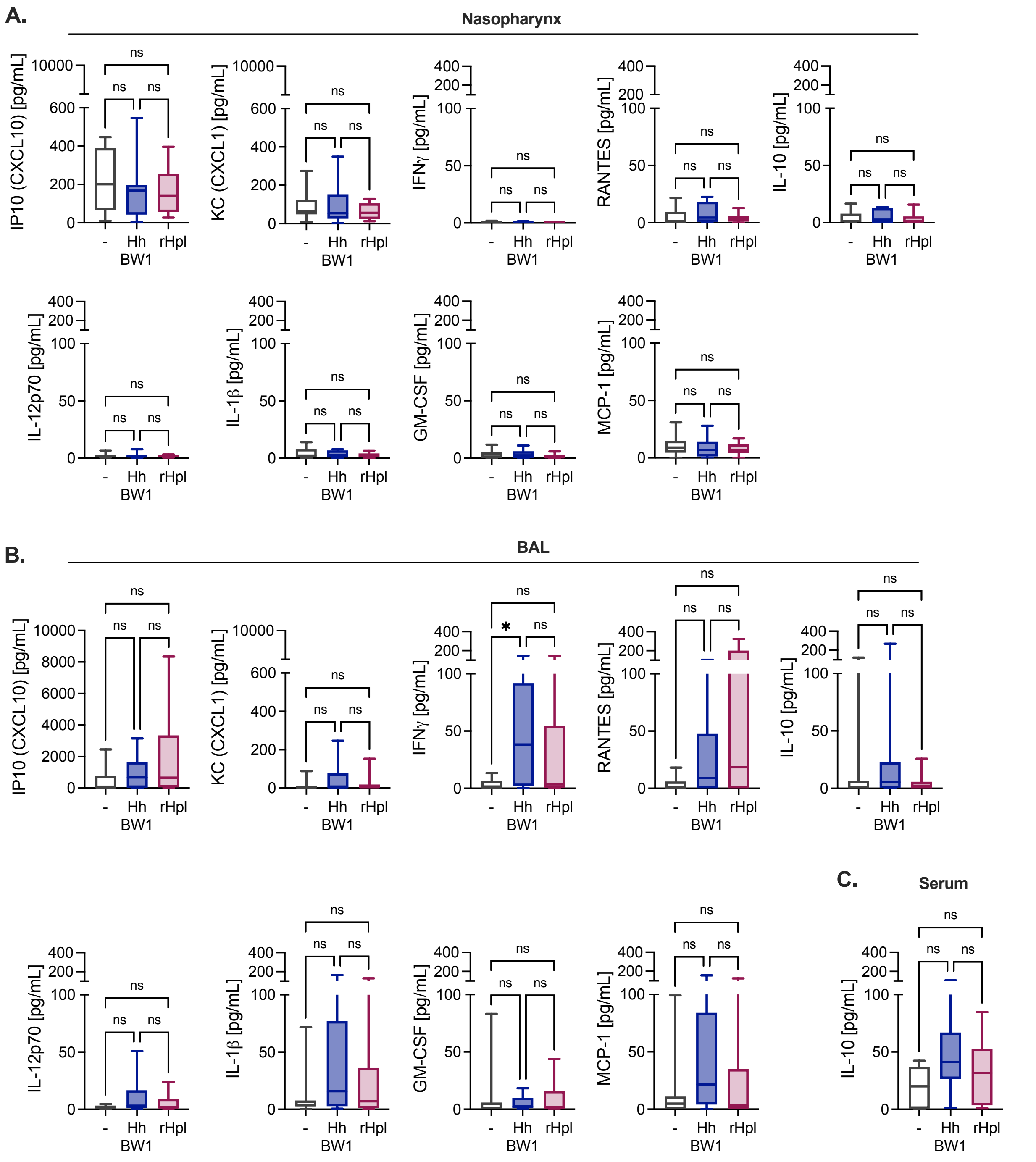

Supplemental Figure 3. Cytokines and chemokines detected in mice infected with NTHi with and without prior exposure to H. haemolyticus or rHpl. A-C Cytokines and chemokines detected 24 hours post-infection with NTHi strain H632 10^8 CFU/mouse i.n. in the nasopharyngeal lavage (A), BAL (B), and serum (C) of mice with

or without (-) pre-exposure to *H. haemolyticus* WT BW1 (Hh BW1) or rHpl (*n* = 12 mice/group). Box boundaries indicate the 25th and 75th percentiles, with a horizontal line representing the median and whiskers indicating minimum and maximum values. Data are displayed as mean ± SEM.

Table I. Score of histopathologic changes in lungs.

Score	Cell Infiltrate	Hemorrhage	Swelling of Alveolar Septa	Lung Damage	Damage Extension
0	Normal amounts of alveolar macrophages. No intra-alveolar inflammatory cells. No bronchiolitis and no inflammatory cells within lumen.	No intra-alveolar red blood cells and/or fibrin	None	Normal lung architecture	No lesions*
1	Any increase in macrophages or inflammatory cells in alveoli and/or bronchioles	Some intra-alveolar red blood cells and/or fibrin	Some / patchy	Minimal change in lung architecture	Focal or small lesions
2	Moderate to severe increase in macrophages or inflammatory cells in alveoli and/or bronchioles	Most of lung with intra-alveolar red blood cells and/or fibrin	Diffuse, but recognizable alveoli	Moderate change in lung architecture with alveoli and bronchioles distinguishable	Large or diffuse lesions
3	Obliterative bronchiolitis and/or lung architecture obscured by inflammatory cell infiltrates		Diffuse and obscuring lung architecture	No recognizable lung structures	

Scored parameters were evaluated on digitized whole slide images with Aperio ImageScope software. *Discrete areas of inflammation and/or architectural disruption. (Adapted from

González LA, Melo-González F, Sebastián VP, et al. Characterization of the Anti-Inflammatory Capacity of IL-10-Producing Neutrophils in Response to *Streptococcus pneumoniae* Infection. Front Immunol. 2021;12:638917. Published 2021 Apr 28. doi:10.3389/fimmu.2021.638917)
